# Dendritic Cell Responses and Function in Malaria

**DOI:** 10.3389/fimmu.2019.00357

**Published:** 2019-03-04

**Authors:** Xi Zen Yap, Rachel J. Lundie, James G. Beeson, Meredith O'Keeffe

**Affiliations:** ^1^Burnet Institute, Melbourne, VIC, Australia; ^2^Department of Medicine, Dentistry, and Health Sciences, The University of Melbourne, Parkville, VIC, Australia; ^3^Department of Biochemistry and Molecular Biology, Biomedicine Discovery Institute, Monash University, Clayton, VIC, Australia; ^4^Department of Microbiology and Central Clinical School, Monash University, Clayton, VIC, Australia

**Keywords:** dendritic cells, malaria, *Plasmodium falciparum*, *Plasmodium vivax*, vaccines

## Abstract

Malaria remains a serious threat to global health. Sustained malaria control and, eventually, eradication will only be achieved with a broadly effective malaria vaccine. Yet a fundamental lack of knowledge about how antimalarial immunity is acquired has hindered vaccine development efforts to date. Understanding how malaria-causing parasites modulate the host immune system, specifically dendritic cells (DCs), key initiators of adaptive and vaccine antigen-based immune responses, is vital for effective vaccine design. This review comprehensively summarizes how exposure to *Plasmodium* spp. impacts human DC function *in vivo* and *in vitro*. We have highlighted the heterogeneity of the data observed in these studies, compared and critiqued the models used to generate our current understanding of DC function in malaria, and examined the mechanisms by which *Plasmodium* spp. mediate these effects. This review highlights potential research directions which could lead to improved efficacy of existing vaccines, and outlines novel targets for next-generation vaccine strategies to target malaria.

## Introduction: Malaria

**Figure 1:**
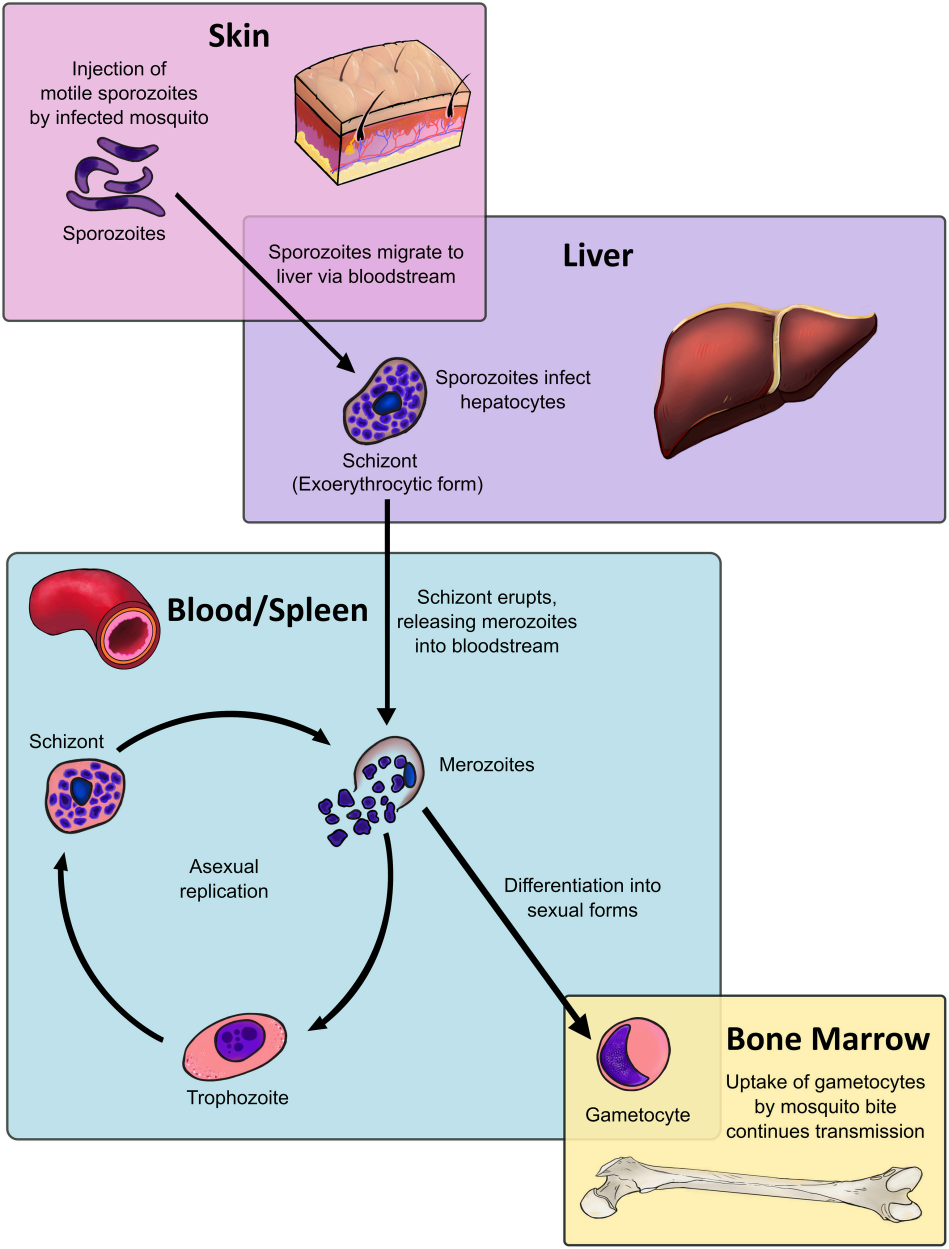
Dendritic cells, located throughout the body at various stages of maturity, interact with all stages of the malaria parasite life cycle within the human host. The *Plasmodium* life cycle encompasses multiple life stages across a range of tissues. The asexual life cycle in the human host begins when mosquitoes inject sporozoites, the highly motile infectious life stage, into the host's skin. The sporozoite migrates to the liver, where it traverses multiple host cells before entering into an exoerythrocytic form. The exoerythrocytic form matures into a multinucleate schizont, which releases merozoites into the bloodstream upon lysis. Merozoites infect host red blood cells and mature into intraerythrocytic life stages known as trophozoites, which are highly metabolically active. After DNA replication the trophozoite will become a blood-stage schizont, which will lyse and release daughter merozoites into the bloodstream, resuming the process. Instead of becoming trophozoites, a fraction of merozoites will instead differentiate into sexual stages known as gametocytes, which sequester in the bone marrow. Only at the end of their maturation process do gametocytes re-enter the bloodstream, where they are taken up by mosquito bite to commence sexual replication in the mosquito host and continue the cycle.

Malaria remains one of the greatest challenges to public health in the developing world. It is caused by infection with the *Plasmodium* species of Apicomplexans, which have a complex life cycle spanning multiple organ sites (Figure 1), facilitated by multiple morphologically and antigenically distinct life stages, and expression of multiple antigens (1–5).

The *Plasmodium* life cycle bridges two hosts: mosquitoes, where sexual replication occurs, and humans, where the parasite undergoes asexual replication. The latter begins when an infected mosquito injects sporozoite-stage parasites from mosquito salivary glands into the skin (Figure 1). A small fraction of sporozoites will travel to the liver, where the sporozoite will traverse hepatic tissue until it locates a suitable hepatocyte. The subsequent exoerythrocytic form will release merozoites into the bloodstream upon rupture (6). *Plasmodium vivax* can also enter a dormant liver stage known as the hypnozoite, which can mature and produce merozoites weeks to years after the initial infection (7, 8). Despite being only 1 μm in size, the merozoite expresses a range of parasite proteins that ligate host red blood cell (RBC) ligands to drive invasion. After invasion the merozoite forms a parasitophorous vacuole in host cells, where it begins to mature into a trophozoite (9).

From 18 to 32 h post-invasion, the trophozoite increases DNA replication and metabolic activity. The mid-trophozoite stage exports various parasite proteins, including those crucial to host pathology, such as the *P. falciparum* erythrocyte membrane protein 1 (*Pf* EMP1) (10). At 34 h post-invasion, the parasite becomes a multinucleate, segmented stage known as the schizont. After 48 h of intracellular maturation and replication, the schizont ruptures, destroying the erythrocyte and releasing parasite metabolites, waste products, and between 16 to 32 daughter merozoites are released into the bloodstream (9), where the cycle will begin afresh.

After 7–15 days in circulation, a small proportion of *P. falciparum* trophozoites will commit to sexual replication, where the process of schizogony is replaced by the formation of sexual stages known as gametocytes (11, 12). Generation of *P. vivax* gametocytes is much faster, with gametocytes being detectable in circulation from 3 days post-infection (13, 14). Gametocytes undergo five maturation stages: stages I-IV preferentially sequester in the bone marrow (BM) and spleen (15–17) while stage V gametocytes re-enter the circulation, where they can be taken up by the bite of infected mosquitoes (18).

The effect of each malaria life stage on host immune function is not well understood, nor are the broader underlying mechanisms of antimalarial immunity. It is frequently observed that individuals living in highly endemic regions develop clinical immunity against symptomatic disease, but generally do not develop sterilizing immunity that completely protects against infection. Antibodies are a crucial component of naturally acquired clinical immunity, as passive transfer of immunoglobulins from malaria immune to non-immune individuals is sufficient to reduce parasitaemia and resolve symptoms (19). Furthermore, clinical immunity appears in most cases to be relatively short-lived and broadly declines in the absence of boosting [reviewed in (20)]. An improved understanding of antimalarial immunity will enable development of future vaccines which can accelerate acquisition of clinical immunity, or better yet, induce sterile immunity.

### Malaria Vaccines

The most advanced malaria vaccine candidate to date is RTS,S, which targets the circumsporozoite protein (CSP) of *P. falciparum*. RTS,S has shown modest efficacy in Phase III clinical trials, with 29 and 36% efficacy in young infants and young children, respectively over 3–4 years, with a booster dose given at 20 months (21). The sub-optimal efficacy of RTS,S and its failure to elicit protective immunity in many recipients is poorly understood (21–23). To elucidate the immunological responses that future malaria vaccines should aim to induce or improve upon, it is vital to understand how different parasite life stages modulate the host immune system. This review focuses specifically on the interactions between malaria parasites and dendritic cells (DCs), sentinel antigen presenting cells of the immune system that are crucial for generating effective immune responses and immunological memory.

### Dendritic Cells

**Figure 2:**
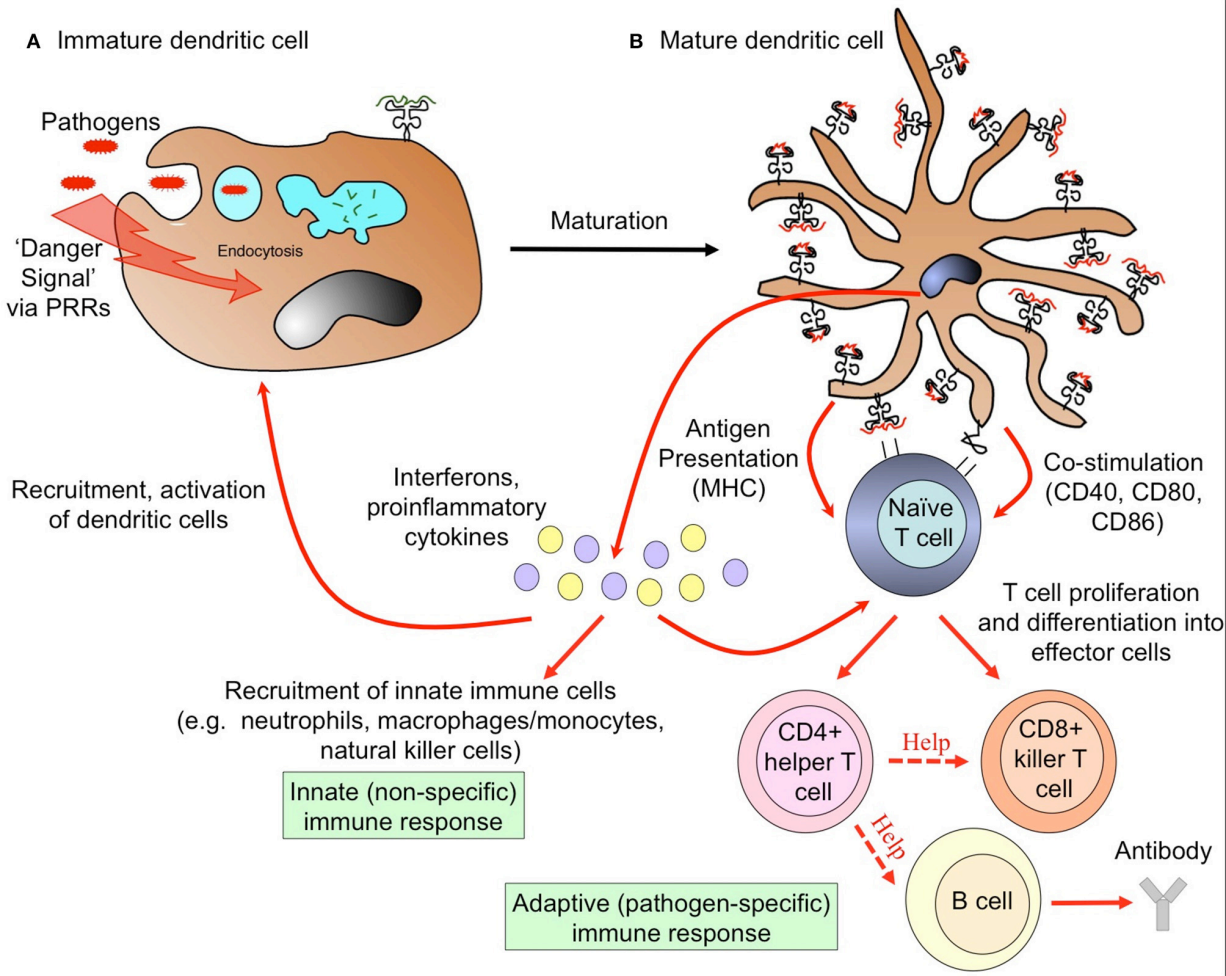
Dendritic cells link innate and adaptive arms of the immune system. **(A)** Uptake of pathogens and recognition of pathogen-associated “danger signals” by pattern recognition receptors (PRRs) triggers dramatic morphological and functional changes in DCs, termed maturation. These changes involve the formation of dendrites, down-regulation of antigen uptake, and redistribution of major histocompatibility complex (MHC) molecules from intracellular endocytic compartments to the cell surface. **(B)** Mature DCs migrate to draining lymph nodes and present information about the invading pathogen in the form of processed peptides loaded onto MHC molecules to naïve T cells. Upregulation of MHC and co-stimulation molecules enables activated DCs to initiate adaptive T and B cell immune responses, the nature of which are determined by the cytokine milieu. This initiates the cascade to an adaptive immune response, leading to clearance of infected cells, and extracellular pathogens. Activated mature DCs also secrete interferons and proinflammatory cytokines that recruit circulating innate immune cells to provide rapid defense against infection.

DCs function as a crucial bridge between innate and adaptive immunity. In a healthy individual, DCs constitute only 1% of all peripheral blood mononuclear cells (PBMC) (24–26), yet they exert potent regulatory effects on both the innate and adaptive immune system (Figure 2). Upon encountering foreign antigens in the presence of pathogen associated molecular patterns (PAMPs), DCs undergo a process of maturation and migrate to the spleen and draining lymph nodes where they interact with pathogen-specific T cells. In addition to presenting antigen via major histocompatibility complex (MHC) surface molecules, DCs express co-stimulatory molecules required for naïve T cell proliferation and differentiation into effector cells, including CD40, CD80 (B7-1), and CD86 (B7-2). Through secretion of cytokines and chemokines, DCs recruit other immune cells and influence the nature of the adaptive T and B cell response, ultimately leading to clearance of infected cells and extracellular pathogens (Figure 2). Crucially, DCs are present at all clinically relevant sites for the development of *Plasmodium* life stages, namely the skin, blood, bone marrow, spleen, and liver (Figure 1).

Based on the expression of CD11c and CD123, human DCs can be broadly classified into plasmacytoid DCs (pDC; Lin^−^HLA-DR^+^CD11c^−^CD123^+^) and conventional DCs (cDC; Lin^−^HLA-DR^+^CD11c^+^CD123^−^) populations. The pDCs are the body's major producers of the anti-viral interferon (IFN)-α, though they constitute only 0.35% of PBMCs (25, 26). These cells are crucial in antiviral responses. The cDCs specialize in priming and presenting antigen to T cells (27), and constitute 0.6% of PBMCs (25, 26). Using the blood dendritic cell antigen (BDCA) markers, it is possible to further differentiate cDC populations into cDC1 (BDCA-3^+^/CD141^+^) and cDC2 (BDCA-1^+^/CD1c^+^) subsets, while pDCs express BDCA-2 (CD303) and BDCA-4 (CD304) (28–30).

Given the central role of DCs in sensing infection and orchestrating immune responses, it is not surprising that many pathogens have evolved immune evasion strategies which specifically target DCs in order to interfere with innate and adaptive immune responses (31–34). Thus, understanding how DCs initiate and maintain effective immune responses against malaria parasites, whilst minimizing detrimental and life-threatening immunopathology, is imperative for vaccine development.

## At the Meeting Points: Sites of DC and *Plasmodium* spp. Interaction

Interactions between DCs and *Plasmodium* parasites occur at every stage of the parasite life cycle within the human host: skin (35), liver (36), and most importantly within the blood and spleen (37), where the majority of host pathology occurs. Recent studies have also revealed that the bone marrow (BM) compartment is a major tissue reservoir for gametocyte development and proliferation of malaria parasites (38–41). Tissue-resident DCs in each of these sites have the potential to endocytose parasite components and initiate the development of specific adaptive immune responses to *Plasmodium* infection. Importantly, DCs in these tissues exist in different maturation states and thus vary in their ability to influence adaptive and innate immune responses and induce inflammatory responses. Within the liver, DCs are thought to induce tolerogenic responses to prevent induction of harmful immunopathology (42, 43), whilst in spleen, DCs propagate strong immune responses, and blood DCs have an intermediate phenotype with a lower capacity for inducing inflammation compared to their splenic counterparts (44).

### Skin and Liver DC Interactions With Sporozoites: Lessons From Murine Models

The skin is the site of first contact between DCs and *Plasmodium* spp. Studies in mice have demonstrated that sporozoites remain in the skin for up to 60 min prior to entering the circulation, after which they lose motility (45). Remarkably, up to 50% of sporozoites become trapped in the dermis, while 30% of those that succeed in entering the circulation enter lymphatic rather than blood vessels (45). Thus, the majority of sporozoites fail to reach the bloodstream and are instead phagocytosed by DCs in the skin-draining lymph nodes, which prime protective CD4^+^ (46–48) and CD8^+^ T cell responses (49, 50). It is likely that a substantial proportion of immunity to sporozoite stages arises predominantly in response to these “failed” sporozoites.

Interestingly, there is some evidence that sporozoites which arrest within the liver may promote induction of limited liver-stage immunity. A murine study demonstrated that apoptosing hepatocytes infected by irradiated sporozoites triggered recruitment of circulating blood DCs to the liver (51). These DCs phagocytosed apoptotic hepatocytes and migrated to lymph nodes, where they induced protective IFN-γ-producing CD8^+^ T cell responses (50).

Importantly, in the above study, infiltrating DCs from the cutaneous lymph nodes initiated immune responses, not liver-resident DCs (50). In humans (52) and mice (53), tissue-resident liver DCs are reportedly less mature than blood DCs, as they are poor at antigen processing and express only low levels of costimulatory markers. While liver DCs in humans are capable of inducing allogeneic T cell responses, they are less effective at this than their blood counterparts, and therefore promote a T cell phenotype that is less responsive to subsequent stimulation (52, 54). When considered in conjunction with their high capacity for IL-10 secretion (52), the liver DC phenotype may be one that promotes a more tolerogenic environment, favorable to sporozoite survival. This could partly explain why sterile immunity rarely occurs in response to natural infection, with tolerogenic liver-resident DCs acting to suppress inflammatory responses which would induce protection. Studies using mouse models with humanized livers have shown promise for investigating *Plasmodium* spp. skin-to-liver transfer (55, 56). In combination with FMS-like tyrosine kinase 3 ligand (Flt3-L)-treated cord blood engrafted humanized mice, which produce large quantities of human DCs similar to those seen in blood (57), combined liver-immune system humanized mice could be a useful avenue to investigate DC involvement in liver-stage immunity.

### The Bone Marrow As a Reservoir for Gametocytes

A similar phenomenon of immune tolerance may occur in the BM, which emerging evidence suggests is a privileged developmental niche for the transmission stages of *Plasmodium*. Autopsy studies have indicated that both *P. vivax* and *P. falciparum* (15, 58–61) gametocytes sequester in the BM, the latter of which is supported by the presence of a *Pf* EMP1 type capable of binding BM endothelium (62). Poor immune responses to parasites in this milieu may be due to tolerogenic potential of the BM microenvironment. There is very little data on BM DCs. One non-human primate study indicated that BM-derived CD123^+^HLA-DR^+^ pDCs had a decreased capacity to express co-stimulatory molecules in response to pathogens relative to blood DCs (63), while CD11c^+^ BM cells in a murine study had a similar capacity for T cell stimulation relative to their blood and spleen counterparts (64). However, it is not clear whether the CD11c^+^ population in the latter study was comprised solely of DCs.

No studies to date have examined how DCs in the BM respond to sequestered parasites, although one murine study has reported that pDCs, present in the BM at frequencies 20 times higher than in the blood or spleen, are the major producers of IFN-α during *P. yoelii 17X YM* infection (65). If the BM is indeed a reservoir for infection, as is suggested by recent primate studies (41), studying whether BM DCs are capable of initiating antimalarial immune responses will be important for achieving elimination.

### Blood and Spleen DC Interactions With Malaria Blood-Stages

Blood-stage parasitaemia provides multiple opportunities for blood and splenic DCs to interact with parasites. The parasite spends the majority of the asexual blood-stage cycle within the host RBC. While *P. vivax* exclusively infects reticulocytes, which express surface MHC and can therefore be cleared by CD8^+^ T cells (66), *P. falciparum* also infects mature RBCs, which do not express surface MHC molecules, thus enabling host immune evasion. Despite this, the blood-stage is an antigenically rich phase of the *Plasmodium* life cycle [reviewed in (67, 68)], affecting a large proportion of host cells and triggering potent inflammatory immune responses that cause most of the symptoms of malaria. Maturation of parasitized RBCs (pRBCs) culminates in lysis of the host RBC, releasing merozoites into the circulation. Merozoites that fail to invade a new RBC will remain in the circulation where they are directly phagocytosed (69) or circulate to the spleen for clearance. The *Pf* EMP1 molecule, which is expressed on the pRBC surface, may play a dual role in this life stage. While it is a prime target for antibodies in naturally acquired immunity (70), one report suggests it may also modulate immune function via binding to CD36 on APCs, including DCs (71). Furthermore, *Pf* EMP1-mediated sequestration in the periphery is long held to be a parasite adaptation aimed at avoiding splenic clearance (72).

DCs play a vital role in initiating and regulating adaptive immunity to blood-stage malaria (73–75). However, there is strong evidence that *Plasmodium* parasites modulate DC maturation and function to interfere with the development of protective immune responses. Data from mouse models indicate that blood-stage infection suppresses both existing and developing liver-stage immunity by inhibiting DC activation (76), and inhibits DCs from responding to subsequently encountered pathogens (77–79). Importantly, murine studies suggest that DCs also play a role in the induction of immune-mediated pathology, including the life-threatening syndrome of cerebral malaria (80, 81). Thus, it is of vital importance that we understand the factors governing the ability of DCs to alter the balance between protection and pathology.

### DCs, Malaria, and Unanswered Questions

The majority of DC-*Plasmodium* interactions in humans have been studied in two ways: (1) studying peripheral blood DCs from currently or previously infected individuals, or (2) measuring DC responses to parasite stimuli *in vitro*. In the first method, DCs were isolated from the blood of individuals who were naturally or experimentally infected with malaria. The surface phenotype and function of these DCs was compared to uninfected controls, either the same individuals prior to or post-infection, or a matched control group (82–102). In the second method, DCs from malaria-naïve individuals were stimulated with *Plasmodium* products to assess the resulting phenotype. The majority of reports which used the latter generated DCs from monocytes *in vitro* using GM-CSF and IL-4 (71, 103–106), while a minority reported responses from *bona fide* DCs from blood (83, 85, 107, 108).

As such, there is limited knowledge about how naïve DC subsets resident in different human tissues and blood respond to *Plasmodium*, and what factors influence this response. This knowledge is vitally important for designing vaccine strategies which specifically enhance the ability of DCs to induce protective responses while limiting induction of immunopathology. Understanding how naïve DC function is altered by *Plasmodium* exposure will provide insight into how DCs are affected in infected individuals, and therefore what vaccine strategies will be required to overcome this altered phenotype.

## Peripheral Blood DC Responses to Natural or Experimental *Plasmodium* Infection

A total of 24 *ex vivo* studies to date have examined how natural or experimental exposure to *Plasmodium* spp. affects the activation phenotype and function of human peripheral blood DCs, in both acute infection and after prior exposure (summarized in Table 1). The following sections analyse these studies in detail, according to species infection.

**Table 1:** *In vivo* exposure to *Plasmodium* species modulates human DC responses.

**References**	**Cohort demographic**	**Transmission intensity (country)**	**DC subset gating strategy (phenotype)**	**Changes in surface molecule expression**	**Serum /plasma cytokines**	**Other effects**
***P. falciparum***						
Urban et al. (82)	Children	Holoendemic (Kenya)	HLA-DR^+^, CD83^+^	Decreased: HLA-DR No change: CD83	Increased: TNF-α IL-10	Decreased DC numbers
Pichyangkul et al. (83)	Hospitalized adults	Mesoendemic (Thailand)	cDC (HLA-DR^+^CD11c^+^) pDC (HLA-DR^+^CD123^+^)		Increased: IFN-α	Reduced numbers of circulating pDC
Breitling et al. (94)	Pregnant women	Holoendemic (Gabon)	cDC (BDCA1^+^) pDC (BDCA2^+^)	No change: HLA-DR*		Decrease in overall DC numbers
Urban et al. (96)	Children	Holoendemic (Kenya)	cDC (CD11c^+^BDCA1^+^, CD11c^+^BDCA3^+^) pDC (CD123^+^BDCA2^+^)	Decreased: HLA-DR (cDC)	Increased: TNF-α IL-10 IL-12	Elevated number of BDCA-3^+^ cDC1 in circulating blood during and after malaria infection Decreased cDC ability to induce allogeneic T cell proliferation
Diallo et al. (97)	Pregnant women	Hypoendemic (Senegal)	cDC (CD11c^+^CD123^lo^) pDC (CD11c^−^CD123^hi^) Less differentiated DC (CD11c^−^CD123^lo^)	No change: CD83* Decreased: HLA-DR*	Increased: TNF-α IFN-γ IL-10	Women who have had malaria have higher percentages of less differentiated DC Decreased circulating pDC in infected pregnant women
Loharungsikul et al. (98)	Hospitalized adults	Mesoendemic (Thailand)	cDC (BDCA1^+^, BDCA3^+^) pDC (BDCA2^+^)	Increased: TLR2 (cDC) Decreased: TLR9 (pDC) No change: TLR4 (cDC)		Decreased fraction of TLR2^+^ cDC in peripheral blood during infection
Fievet et al. (109)	Pregnant women	Mesoendemic (Benin)	cDC (BDCA1^+^, BDCA3^+^) pDC (BDCA2^+^)	Increased: HLA-DR (BDCA1, BDCA2) No change: CD86* HLA-DR (BDCA3)	No change: TNF-α IFN-γ IL-10 IL-12 IL-6 MIP-1α	Increased percentage of HLA-DR positive BDCA2^+^ cells during infection Decreased absolute number of all DCs from women with ≥3 pregnancies Increased pDC number in women with ≥3 pregnancies
Gonçalves et al. (99)	Clinic admission	Hypo- to mesoendemic (Brazil)	cDC (HLA-DR^+^CD11c^+^) pDC (HLA-DR^+^CD123^+^)	No change: CD86*	Increased: IFN-γ TNF-α IL-10	Decreased cDC number Decreased total DC number
Arama et al. (100)	Children	Mesoendemic (Mali)	cDC (HLA-DR^+^BDCA1^+^, HLA-DR^+^BDCA3^+^, HLA-DR^+^CD16^+^) pDC (HLA-DR^+^BDCA2^+^)	Decreased: HLA-DR* CD86 (pDC)	Decreased: IFN-γ	Increased BDCA-2^+^ pDC and BDCA-3^+^ cDC1 populations in peripheral blood Impairment of TLR signaling in DC during malaria results in more severe clinical symptoms
Ibitokou et al. (101)	Pregnant women	Mesoendemic (Benin) Holoendemic (Tanzania)	cDC (HLA-DR^+^BDCA1^+^) pDC (HLA-DR^+^BDCA2^+^)	Decreased: HLA-DR (cDC) Increased: CD86 (pDC)		Decreased pDC and cDC fraction in peripheral blood
Guermonprez et al. (102)	Children	Holoendemic (Kenya)	cDC (CD11c^+^BDCA1^+^, CD11c^+^BDCA3^+^) pDC (CD123^+^BDCA2^+^)		Increased: Flt3L	Increased BDCA-3^+^ cDC1 fraction in peripheral blood Increased CD8^+^ T cell activation in peripheral blood
Pinzon-Charry et al. (84)	Infected adults	Holoendemic (Papua)	cDC (HLA-DR^+^CD11c^+^) pDC (HLA-DR^+^CD123^+^) iDC (HLA-DR^+^CD123^−^CD11c^−^)	Decreased: HLA-DR CD83 CD86	Increased: TNF-α IFN-γ IL-2 IL-4 IL-6 IL-10	Decreased pDC and cDC fractions Increased fraction of immature DC Increased DC apoptosis Decreased ability to induce T cell proliferation Decreased antigen uptake
**Restimulation of DCs from naturally** ***P. falciparum*****-infected individuals**^†^						
Fievet et al. (109)	Pregnant women	Mesoendemic (Benin)	cDC (BDCA1^+^, BDCA3^+^) pDC (BDCA2^+^)	No change: HLA-DR (cDC)	Increased: IFN-γ TNF-α IL-10 No change: IFN-α IL-6 IL-12 MIP-1α	Increased production of proinflammatory cytokines by women ≤ 25 years independent of gravidity
Götz et al. (85)	Adults	Holoendemic (Mali)	cDC (HLA-DR^+^BDCA1^+^, HLA-DR^+^BDCA3^+^, HLA-DR^+^CD16^+^)	Increased: HLA-DR CD86 No change: CD40 CD80	Increased: CCL2 CXCL9 CXCL10 No change: IL-1β IL-6 IL-10 TNF-α CCL5	
***P. vivax***						
Jangpatarapongsa et al. (86)	Hospitalized adults	Mesoendemic (Thailand)	cDC (HLA-DR^+^CD11c^+^) pDC (HLA-DR^+^CD123^+^)		Increased: IL-10	Decreased fraction of cDC and pDC during infection Increased numbers of FOXP3^+^ T_REG_ during infection
Gonçalves et al. (99)	Clinic admission	Hypo- to mesoendemic (Brazil)	cDC (HLA-DR^+^CD11c^+^) pDC (HLA-DR^+^CD123^+^)	*Decreased*: CD86*	Increased: TNF-α IL-10	Increased pDC fraction Decreased cDC number Decreased total DC number
Pinzon-Charry et al. (84)	Infected adults	Holoendemic (Papua)	cDC (HLA-DR^+^CD11c^+^) pDC (HLA-DR^+^CD123^+^) iDC (HLA-DR^+^CD123^−^CD11c^−^)	Decreased: HLA-DR CD83 CD86	Increased: TNF-α IFN-γ IL-2 IL-4 IL-6 IL-10	Decreased pDC and cDC fractions Increased fraction of immature DC Increased DC apoptosis Decreased T cell proliferation Decreased antigen uptake
***P. falciparum*** **and** ***P. vivax*** **coinfection**						
Gonçalves et al. (99)	Clinic admission	Hypo- to mesoendemic (Brazil)	cDC (HLA-DR^+^CD11c^+^) pDC (HLA-DR^+^CD123^+^)	No change: CD86*	Increased: TNF-α IL-10	Decreased cDC fraction Increased pDC fraction
Kho et al. (87)	Children, adults, hospitalized children, and adults	Holoendemic (Papua)	cDC (HLA-DR^+^BDCA1^+^, HLA-DR^+^BDCA3^+^) pDC (HLA-DR^+^BDCA2^+^)	Increased: HLA-DR* Decreased: HLA-DR (cDC)		Increased pDC fraction during asymptomatic *P. vivax* but not *P. falciparum* infection Decrease in BDCA-1^+^ cDC2 fraction during asymptomatic infection with either species or during uncomplicated malaria
Kho et al. (88)	Adults	Holoendemic (Papua)	cDC (HLA-DR^+^BDCA1^+^, HLA-DR^+^BDCA3^+^) pDC (HLA-DR^+^BDCA2^+^)	Decreased: HLA-DR		Decreased numbers of circulating pDC and BDCA-1^+^ cDC2 during symptomatic infections but not during subpatent infections Reduced CD4^+^ T cell proportion Decreased proportion of activated and resting T_REG_
**Controlled human malaria infection with** ***P. falciparum***						
Woodberry et al. (89)	Healthy adult males	N/A (CHMI)	cDC (HLA-DR^+^CD11c^+^) pDC (HLA-DR^+^CD123^+^)	Decreased: HLA-DR (pDC)	No change: TNF-α IL-6 IL-10 IL-12	Increased DC apoptosis Decreased overall DC numbers Decreased phagocytic activity
Teirlinck et al. (90)	Healthy adults	N/A (CHMI)	cDC (HLA-DR^+^BDCA1^+^, HLA-DR^+^BDCA3^+^) pDC (HLA-DR+BDCA2^+^) CD16^+^ DC (HLA-DR^+^CD16^+^ CD14^−^)	Increased: HLA-DR (CD16^+^, pDC) CD86 (CD16^+^) CD16 (CD16^+^, BDCA-1^+^, pDC) CD1c (CD16^+^, BDCA-1^+^, pDC) No change: HLA-DR (cDC) CD86 (cDC, pDC)		Increased expression of BDCA-1 and CD16 on all subsets except BDCA-3^+^ cDC1 Increased CD1c/BDCA-1 and CD16 expression on monocytes after treatment
Loughland et al. (91)	Healthy adults	N/A (CHMI)	cDC (HLA-DR^+^CD11c^+^ BDCA-1^+^)	Decreased: HLA-DR CD86	Increased: TNF-α No change: IL-12	Increased DC apoptosis Decreased antigen uptake Decreased overall DC numbers
Loughland et al. (92)	Healthy adults	N/A (CHMI)	pDC (HLA-DR^+^CD11c^−^CD123^+^)	Decreased: HLA-DR No change: HLA Class I	No change: TNF-α Increased: IFN-α	Reduced circulating pDC at and 24 h after peak parasitaemia Increased caspase-3 expression in pDCs Increased expression of *NLRC5, C14orf119* and *TSG101* Decreased expression of *DMBT1, AREGB, RNF139, CRYM*, and *BAG3*
Loughland et al. (93)	Healthy adults	N/A (CHMI)	CD16^+^ DC (HLA-DR^+^CD14^−^CD11c^+^BDCA-1^−^CD16^+^CD86^+^)	Decreased:CD16 Increased: HLA-DR CD86	Increased: TNF-α IL-10	Increased proportion of CD16^+^ DCs among all CD11c^+^ DCs Non-parasite-specific loss of CD16 when DCs are in culture
**Controlled human malaria infection with** ***P. vivax***						
Woodberry et al. (95)	Healthy adults	N/A (CHMI)	cDC (HLA-DR^+^CD11c^+^ BDCA1^+^, HLA-DR^+^CD11c^+^ BDCA3^+^) pDC (HLA-DR^+^CD123^+^) CD16^+^ DC (HLA-DR^+^CD11c^+^ CD16^+^)	Decreased: HLA-DR (cDC) CD123* No change: HLA-DR (pDC)	Increased: IFN-γ	Reduced circulating pDC, BDCA-1^+^ and BDCA-3^+^ DC Increased caspase-3 expression in pDC, CD16^+^, and BDCA-1^+^ cDC Increased numbers of activated T_REG_ during infection Increased indoleamine 2,3-dioxygenase metabolism drives T_REG_ differentiation

Transmission intensity, reported intensity of transmission in the catchment area at the time of sample collection; DC subset gating strategy, subsets and markers used to define the subsets which were examined in the study; changes in surface molecule expression, change in activation marker expression after exposure to Plasmodium, relative to healthy controls; serum/plasma cytokines, cytokines assayed for in serum or plasma, change measured relative to healthy controls (no stimulation/no exposure); other effects, other observed changes in cell populations, numbers, or function. ^*^All DC subsets affected.

† *Surface molecule expression and cytokine secretion for DCs in these studies were measured in supernatants of purified DC cultures after restimulation with pRBCs (85) or TLR ligands (109), as opposed to direct measurement of DC phenotypes from whole blood*.

### DC Phenotypes and Responses During *P. falciparum* Infection

*Plasmodium falciparum* is responsible for a high burden of morbidity and mortality in pregnant women and children, and can cause severe and fatal disease outcomes including cerebral malaria, miscarriage, and multiple organ failure (110). Infected persons typically present to hospital when blood-stage infection becomes symptomatic, which can occur nine to 30 days after the initial infection (111). Classifying malaria cases as mild/uncomplicated vs. severe is based on specific clinical features, including but not limited to coma, haemoglobinuria, vital organ dysfunction, or respiratory distress (110). The majority of *ex vivo* studies have been carried out in settings of high *P. falciparum* transmission, focusing on the phenotype and function of DCs in high-risk groups including children and pregnant women (Table 1).

#### DCs and *P. Falciparum* in Children in High-Transmission Settings

In a DC study comparing infected children to non-infected controls in a holoendemic setting, Kenyan children hospitalized with mild vs. severe malaria exhibited decreased HLA-DR expression on DCs and reduced DC numbers in circulating blood, regardless of disease severity (82). A subsequent study which followed children during malaria and after treatment showed that malaria specifically decreased HLA-DR expression on cDC but not pDC subsets, and reduced the ability of DCs to induce allogeneic T cell proliferation in mixed leucocyte reactions (MLR) (96). Furthermore, infection correlated to an increase in absolute numbers of circulating BDCA-3^+^ cDC1s. Importantly, these effects of *P. falciparum* on DC phenotype and function were still observed 14 days after hospital discharge and curative treatment (96), suggesting that malaria-induced immunosuppression can persist for some time after parasite clearance.

A subsequent study was conducted in Mali, another holoendemic setting, where DC function was compared between infected and non-infected children from the Fulani and Dogon ethnic groups. DCs from children aged 2–10 years displayed reduced HLA-DR expression after malaria exposure (100). Infection was also associated with increased proportions of circulating BDCA-2^+^ pDC and BDCA-3^+^ cDC1 populations, with reduced CD86 expression in the former (100). In this study genetic differences were proposed to play a role in clinical outcomes of *P. falciparum* infection due to differences in cytokine production between the 2 ethnic groups, with PBMCs from Dogon children displaying significantly impaired cytokine production, correlating with more severe fever and higher parasitaemia (100). These responses could be attributed in part to reduced DC function, including a reduction of pDC-derived IFN-α production in response to TLR9 ligands.

More recently, Guermonprez et al. reported that children with malaria, regardless of disease severity, had an increased frequency of the BDCA-3^+^ cDC1 population (102). This correlated with increased serum concentrations of the DC growth factor Flt3-L that preferentially increases pDC and cDC1 *in vivo* (112, 113). During malaria, Flt3-L is produced by mast cells in response to uric acid metabolism by *Plasmodium* parasites (102).

Together, these studies suggest that malaria in children in high-transmission settings negatively impacts DC activation marker expression and modulates DC function. The low activation status of peripheral DCs may be due to sequestration of activated DCs in affected tissues. Moreover, an increased number of circulating BDCA-3^+^ cDC1s appears to be a common feature of malaria in this setting. Urban et al. also showed that DC dysfunction persisted after the resolution of malaria (96), leaving these individuals vulnerable to co-infections. The apparent contradiction between reduced DC numbers in the first study (82) and elevated numbers of BDCA-3^+^ cDC1s in the second study (96) is likely due to more sophisticated gating strategies in the latter, enabling discrimination of individual DC subsets (96), rather than classifying all HLA-DR^+^ cells as DCs (82). Rigorous and well-defined flow cytometry gating strategies that use an appropriate combination of antibodies to DC subset-specific surface markers are imperative for DC research and may help to resolve some of the apparent discrepancies in the literature.

#### DCs and *P. Falciparum* in Pregnancy in High-Transmission Settings

Four studies evaluating changes in DC populations in infection during pregnancy have yielded conflicting results. Two studies, one from Gabon (94) and one from Benin and Tanzania (101), observed that overall DC numbers were decreased in pregnant women infected with *P. falciparum* compared to uninfected matched pregnant controls, while a study from Senegal (97) reported a decrease in the pDC population only relative to non-pregnant uninfected controls. Another study from Benin (109) did not observe any difference in DC numbers between infected and non-infected pregnant women. Changes in surface activation marker expression varied across studies (Table 1).

Again, different gating strategies may underlie some of the differences observed between these studies. Simply gating on CD123^+^ or CD11c^+^ populations may run a risk of false positives if isolation and lineage staining is not extensive enough. Use of cord blood (94, 97) or placenta-derived (97) DCs may also contribute to phenotypic differences between these DCs and peripheral blood DCs, due to the unique microenvironments of these pregnancy-associated tissues. Gravidity can also be an important contributing factor. Since primigravid women are at the highest risk of severe inflammatory disease [reviewed in (114)], the proportion of women in their first pregnancy should always be accounted for in immunological studies. Inclusion of pregnant non-infected controls is also imperative to determine whether pregnancy itself is a confounding factor affecting DC function during malaria.

#### Function of DCs From Naturally Exposed Individuals

Three studies of adults with symptomatic malaria carried out in Thailand (98), Brazil (99), and Papua (84) provide insights into how *P. falciparum* immunity develops in lower-transmission settings. Within the Thailand cohort, activation marker expression was not assessed, but circulating numbers of pDCs were significantly reduced in both mild and severe malaria compared to healthy controls. IFN-α levels in the serum increased (83), but it was not established whether this directly correlated with pDC function. The percentage of immature HLA-DR^+^CD11c^−^CD123^−^ cells in circulation increased, while the fractions of circulating CD11c^+^ cDCs and CD123^+^ pDCs were decreased. DCs from infected participants were apoptotic (upregulated the apoptotic marker Annexin-V) and were defective at antigen uptake and induction of naïve T cell proliferation in allogeneic T cell activation assays (84). All three cohorts were recruited via clinical admissions, which self-selects for individuals with lower pre-existing immunity and perhaps a more naïve phenotype.

In short, it appears that while impairment is more pronounced in high-transmission settings due to frequent re-infection and higher overall parasite burden, downregulation of DC function is a common feature of malaria. Considering that malaria induces potent inflammation, this DC phenotype may therefore be comparable to what is seen in other inflammatory diseases such as bacterial sepsis (115), HIV (116), or HCV (117). In these patients it is also common to observe reductions in circulating DC numbers (115, 116) and reduced HLA-DR (117) or CD86 (115, 117) expression. Persistent systemic inflammation may therefore explain this reduction in DC function in naturally malaria-infected persons. Again, more rigorous classification of cDC1, cDC2, and pDCs may clarify some of the discrepancies amongst different reports.

#### Stimulation of DCs From Naturally Exposed Individuals

In a study examining DC responses to TLR stimulation after natural *P. falciparum* infection, DCs from naturally exposed pregnant women in Benin were collected from cord blood (109). Whole PBMC cultures were stimulated with TLR4 ligand LPS, TLR3 ligand polyinosinic:polycytidylic acid (polyI:C), or TLR9 ligand CpG-A ODN to stimulate BDCA-1^+^ cDC2, BDCA-3^+^ cDC1, or pDCs, respectively, due to the high expression of each TLR on these specific DC subsets (118). Synthetic hemozoin prepared from haemin chloride was also used for DC stimulation. There was no difference in HLA-DR expression between infected and non-infected women upon stimulation with either TLR ligands or hemozoin. PBMCs from infected women produced more TNF-α and IL-10 in response to CpG-A stimulation, more IFN-γ in response to polyI:C, and more TNF-α in response to hemozoin relative to non-infected women (109).

Only one study to date has stimulated DCs from naturally exposed individuals using pRBCs (85). DCs were purified from the blood of adults from a highly endemic region in Mali at the end of the transmission season and DC activation was compared to that in naïve controls. All exposed individuals were PCR-negative for infection at the time of enrolment (85). When stimulated with pRBCs at a ratio of 3 pRBCs per DC, DCs from these individuals upregulated expression of HLA-DR and CD86 and expressed CCL2, CXCL9, and CXCL10, but did not produce any IL-1β, IL-6, IL-10, or TNF-α (85). In Section 4, this review outlines how a lack of cytokine secretion is commonly observed in *in vitro* studies of *bona fide* DC, and therefore should not necessarily be considered a sign of DC suppression. However, it is interesting that when DCs isolated from malaria-exposed individuals were stimulated with pRBCs following cessation of high malaria transmission (85), DCs could express an activatory surface phenotype in response to stimulation. Thus, it may be that sustained reductions in transmission allow restoration of DC function.

#### TLR Modulation in DCs by *P. falciparum*

Only one study to date has investigated the ability of *P. falciparum* to modulate TLR expression on DCs as a potential mechanism of immune suppression (98). In this study, individuals with severe or mild *P. falciparum* infection exhibited increased TLR2 expression on cDCs but decreased TLR9 expression on pDCs, with no observable change in TLR4 expression (98) compared to healthy controls. The severity of infection did not impact these changes in TLR expression. Moreover, the fraction of TLR2^+^ DCs in the periphery decreased during infection (98). TLR2, TLR4, and TLR9 have all been implicated in sensing of *Plasmodium*-derived “danger signals.” Namely, TLR2 and TLR4 recognize glycophospholipid (GPI) anchors for merozoite surface proteins (119), and TLR9 detects *Plasmodium* DNA (120). As this is the only study to assess TLR expression profiles during *Plasmodium* infection, it is unclear whether this effect is a common feature of malaria. Nevertheless, it suggests that even low-level *Plasmodium* infections can modulate host responses by downregulating the signals required for APC activation.

### The Effects of Natural *P. vivax* Infection on DC Phenotype and Function

*Plasmodium vivax* is the second major malaria pathogen. It inhabits a broader geographical range than *P. falciparum*, posing a risk to more than 3.2 billion individuals worldwide (121). Its pathogenic potential is enhanced by its ability to become a latent hypnozoite in the liver (7), but as it exclusively infects reticulocytes (122), it is difficult to maintain in culture and remains relatively understudied. Immunity to *P. vivax* has primarily been studied in symptomatic persons who present to healthcare. As the geographical ranges of *P. vivax* and *P. falciparum* transmission overlap, it is often difficult to exclude the immunological impact of prior *P. falciparum* exposure. Nonetheless, it is possible to describe the acute effects of *P. vivax* single-species infection, even though an individual's infection history may be unclear, if diagnosis is sufficiently rigorous. The gold standard for species-specific diagnosis is PCR. However, in resource-poor settings rapid diagnostic tests are typically used.

Due to the paucity of studies from *P. vivax*-exposed individuals it is difficult to conclude the effects of *P. vivax* malaria on DC function. DC numbers decreased during infection, both as a fraction (84, 86) and as total numbers (99). In the latter study, the pDC fraction was increased while cDC numbers decreased (99). Another study observed a decrease in both pDC and cDC fractions, as well as increased DC apoptosis (84). *Plasmodium vivax* malaria has also been reported to down-regulate CD86 expression on DCs (84, 99).

### The Effect of Mixed *Plasmodium* Infections on DC Function

Phenotypic analyses of peripheral blood DC from individuals co-infected with two *Plasmodium* spp. support similar reductions in overall DC numbers as seen in individuals experiencing single infections (87, 88, 99). However, it is not yet known whether this correlates to impairments in DC function. A study from Gonçalves et al. in a mesoendemic area of Brazil found that asymptomatic individuals infected with both *P. falciparum* and *P. vivax* had decreased circulating cDCs but increased circulating pDCs (99). Studies in a holoendemic region of Papua found that pDC fractions increased during asymptomatic *P. vivax* but not *P. falciparum* infection, with pDC and BDCA-1^+^ cDC2 fractions decreasing during acute infection with either species (87, 88). No changes were observed in the BDCA-3^+^ cDC1 fraction in children or adults during acute or asymptomatic infection with either species (87, 88), in contrast to the findings in African cohorts (96, 100, 102). HLA-DR expression on DCs was increased during asymptomatic *P. vivax* infection (87), but decreased during acute mixed or single-species infections (87, 88).

It is interesting that HLA-DR expression on DCs was positively correlated with parasitaemia in children with asymptomatic *P. vivax* infection, but negatively correlated with parasitaemia in adults with asymptomatic *P. falciparum* infection (87). Thus, it may be that the two major pathogenic *Plasmodium* species polarize the immune system in different ways. This data also suggests fundamental differences in how childrens' and adults' DCs respond to *Plasmodium* exposure—an important factor to keep in mind considering the at-risk populations for either species.

### Insights From Controlled Malaria Infection Models

#### Controlled Human Malaria Infection With *P. falciparum*

The development of a controlled human malaria infection model (CHMI) has produced valuable insights into antimalarial immunity. In one CHMI model which has been used to study DC in malaria, healthy volunteers who are typically malaria-naïve were inoculated with an ultra-low (<180) or low (1,800) dose of *P. falciparum* pRBCs thawed from a pre-prepared biobank. Atovaquone/proguanil or artemether/lumefantrine treatment was administered 6 days post-infection (ultra-low-dose group) or when parasitaemia reached 1,000 parasites per mL (low-dose group). Despite the low parasite biomass of the inoculum in the low-dose group, an estimated 20 times lower than the number of merozoites released from an infected hepatocyte after sporozoite replication (123), DC numbers were significantly decreased in the low-dose group due to increased DC apoptosis (89). Intriguingly, infection-induced apoptosis appeared to be exclusive to HLA-DR^+^ cells, including DCs. Furthermore, the decrease in DC numbers coincided with the peak of symptomatic malaria, and while cDC numbers recovered to pre-infection levels after drug treatment, pDC numbers remained at 47% of baseline 60 h post-cure (89). HLA-DR expression on pDCs was also impaired. Importantly, DCs from the low-dose group displayed impaired phagocytosis, which persisted for 36 h after drug cure. In contrast, the ultra-low-dose group experienced no symptoms and no DC impairment (89). This study suggests that a certain parasite biomass is required for functional impairment of DCs. However, since the ultra-low-dose group were treated prior to development of symptoms, it is unclear whether an ultra-low dose is sufficient to induce immunity that can control sub-symptomatic parasitaemia, or whether immune impairment would have eventuated if parasitaemia had been allowed to develop.

##### Function of pDCs and BDCA-1^+^ cDC2s during CHMI

A second controlled infection study from Loughland et al. utilized a similar low- (1800 pRBCs) and ultra-low (150 pRBCs) dose to more closely study BDCA-1^+^ cDC2 activation (91) and pDC function (92) after controlled infection. Unlike the prior study, patients were treated upon reaching a parasitaemia of 1000 pRBCs per mL, regardless of initial parasite inoculum. Importantly, both groups experienced a decrease in HLA-DR expression on BDCA-1^+^ cDC2s that coincided with peak parasitaemia but also persisted 24 h after drug treatment (91). However, only the high-dose group exhibited decreased DC numbers, increased DC apoptosis, and reduced phagocytic capacity relative to baseline (91, 92). A positive association was also observed between phagocytic activity and HLA-DR expression at peak parasitaemia (91).

The ability of DCs to respond to TLR stimulation after exposure to malaria was also evaluated in these studies (91) by restimulating DCs taken from participants during peak parasitaemia. Interestingly, the BDCA-1^+^ cDC2 from individuals in the high-dose group were impaired in their capacity to upregulate HLA-DR and CD86 in response to stimulation with TLR1/2, TLR4, and TLR7 ligands or whole pRBCs. This impairment was DC-specific, as monocytes' capacity for activation marker expression was unaltered by malaria exposure (91). In contrast, pDCs restimulated with TLR7 and TLR9 ligands upregulated expression of HLA-DR, CD123, and IFN-α, and upregulated CD86 in response to TLR7 stimulation (92). The cDC1 subset was not examined in these studies. These results were similar to TLR stimulations of cord blood DCs from pregnant women, where CpG-A stimulation of pDCs showed enhancement of cytokine production in infected individuals (109), though caution must be taken when comparing naïve CHMI participants to naturally-exposed pregnant women in Benin.

Together, these studies suggest that a single infection is sufficient to impair cDC function, while pDC function is more resilient. As discussed further on, this highlights a need to further study pDC function during malaria and the potential role of this subset in immunopathology.

##### CD16^+^ DC function in CHMI

The CD16^+^ DC subset's status as a steady-state DC rather than a monocyte subset that acquires DC-like characteristics during inflammation remains unclear (27, 124, 125). Improved strategies for distinguishing “true” CD16^+^ DCs from CD16^+^CD14^−^ monocytes have not yet been established, although a recent single-cell RNAseq study highlighted a population of BDCA-1^−^BDCA-3^−^CD16^+^ cDCs that is transcriptomically distinct from monocytes (126). However, two studies have examined the role of CD16^+^ “DCs” in malaria, both in CHMI. Both studies observed that relative to pre-CHMI levels HLA-DR and CD86 expression in these DCs increased after curative treatment (90, 93) and 24 h prior to peak parasitaemia (93). At peak parasitaemia CD16^+^ DCs had an increased ability to spontaneously produce TNF-α, IL-10, and IL-12. CD16^+^ DCs collected at peak parasitaemia and restimulated with pRBCs expressed higher levels of IL-10 relative to baseline (93). When restimulated with TLR1/2 or TLR4 ligands, these CD16^+^ DCs produced high levels of TNF-α and moderate amounts of IL-10 and IL-12. When restimulated with TLR7 ligands, the CD16^+^ DCs produced TNF-α only (93). While caution must be taken in ascribing *bona fide* DC status to the CD16^+^ DCs, these studies indicate that these cells are activated during infection and in the highly inflammatory environment post-treatment. Their high production of both TNF-α and IL-10, which may aid in killing or suppression of DCs, respectively, suggest that they could be major contributors to DC modulation, including that seen many days post-treatment and clearance of infection (96).

#### CHMI With *P. vivax*

Due to the technical difficulty of maintaining *P. vivax* in continuous culture, to date only one CHMI has been published using *P. vivax* (95). In this study, peripheral DC numbers were significantly reduced during acute infection relative to baseline, though this was concurrent with an overall reduction in circulating PBMC (95). All subsets (BDCA-3^+^ cDC1s, BDCA-1^+^ cDC2s, pDCs, and CD16^+^ cDCs) upregulated caspase-3 during acute infection and after treatment, suggesting that the reductions in DC numbers in the periphery could also be due to increased apoptosis. Overall, DC impairment by *P. vivax* CHMI was largely similar to what was observed with *P. falciparum* (89, 91); HLA-DR expression on BDCA-1^+^ cDC was reduced during acute infection and 24 h after treatment (95).

### *Ex vivo* DCs in *Plasmodium* Infection: What Do We Know?

In summary, *Plasmodium* infection can result in reduced DC numbers in the periphery, both as an absolute number (89, 91, 94, 99) and as a proportion of total leucocytes (82, 97, 101), reportedly due to increased DC apoptosis (84, 89, 91). DC capacity for phagocytosing antigen is also decreased (89, 91), which correlates with DC activation (127), yet their ability to induce T cell proliferation in allogeneic T cell stimulation assays is impaired (84, 89, 96). HLA-DR expression is generally decreased (87–89, 91, 92, 95–97, 100, 101), with some variability between DC subsets (Table 1). It is not clear whether the reduction in HLA-DR is due to an increase in new immature DCs in the circulation, or direct downregulation by parasites. There is little consensus regarding other markers: reports on CD83 (84, 97) and CD86 expression are contradictory, though CD86 tends to be elevated on pDCs and decreased on DCs as a total population (83, 84, 91, 100, 101).

It is also unclear whether the decrease in the number of circulating DCs is due to cell death, as suggested by the upregulation of caspase-3 (89, 91, 95) or annexin V (84), or due to increased migration to lymphoid tissues. Decreased DC numbers in both natural and experimental infection, however, coincided with increased serum levels of IL-10 (82, 84, 86, 96, 97, 99) and TNF-α (82, 84, 91, 96, 97, 99), indicating a potential cytokine-mediated mechanism of DC loss. One subset in particular defied this trend: proportions of BDCA-3^+^ cDC1s were increased during *P. falciparum* infection (96, 100, 102), and remained elevated for some time after acute infection (96). The BDCA-3^+^ cDC1 subset is associated with the initiation of CD8^+^ killer T cell responses and the secretion of IL-12 (128). It is likely that increases in serum Flt3-L lead to increased numbers of these DC in the periphery during infection, but these circulating DC do not appear to be capable of inducing functional responses. Further complicating the matter, the BDCA-3^+^ cDC1 subset is not elevated in single or mixed infections from Papua, where transmission intensity is comparable (87, 88).

Overall, the different methods and markers that have been used to study DCs in this variety of settings makes it difficult to clearly define universal parameters of DC loss of function. It is possible that the DC downregulation described in these studies is a feedback loop promoting regulatory mechanisms in the face of severe malaria-induced inflammation, and that DC downregulation in malaria is not necessarily detrimental to host survival. However, the presence of functional DCs is required for effective vaccine responses, and it is still not clear how malaria-induced DC downregulation affects survival to other pathogens. There is an overall need to understand how these DC phenotypes correlate to clinical outcomes, or at minimum, how malaria directly affects DC function. It will be important to clarify whether DC downregulation during natural infection translates to suppression, namely loss of generalized immune function against non-malaria pathogens or inflammatory stimuli.

In light of this, *in vitro* studies of DC function are vital for three purposes: (1) clarifying the phenotype of DC suppression, (2) determining precisely how malaria modulates DC function, and (3) identifying whether this is through direct interaction with DCs or indirectly through soluble mediators, including cytokines such as TNF-α.

## Defining the Interactions between DCs and *Plasmodium* spp. *in vitro*

To date, relatively few studies have investigated direct interactions between *Plasmodium* spp. and human DCs *in vitro*. The majority of these studies have examined the responses of human monocyte-derived DCs (moDCs), since they can be easily generated in large numbers from CD14^+^ PBMCs or BM monocytes by co-culture with GM-CSF ± IL-4 (129, 130). MoDC are themselves heterogeneous and contain cells with a cDC-like phenotype with high expression of MHC class I and II, BDCA-1, CD40, CD80, and CD11c (129), and macrophage-like cells (131). Transcriptomic analysis indicates that moDC are highly distinct from blood CD16^+^, cDC2 (BDCA-1^+^), and cDC1 (BDCA-3^+^) cDC subsets and therefore do not accurately represent the diversity of DC populations or their functions *in vivo* (124). Other recent findings indicate that monocyte-derived inflammatory DCs in humans are more similar to macrophages than to *bona fide* DCs [reviewed in (27, 125)]. Thus, moDCs may not be a representative model for investigating *bona fide* human DC responses. These caveats must be considered when interpreting the data from *in vitro* studies (summarized in Table 2).

**Table 2:** DC responses after *in vitro* exposure to *P. falciparum*.

**References**	**DC type**	**Parasite strains**	**Stimulus type**	**Post-stimulation phenotype**	**Cytokines detected**	**Other effects**	**Proposed mechanism of action**
Monocyte-derived DCs*							
Urban et al. (103)	Monocyte-derived	ITO/A4 ITO/C24 MC T9/96	Whole trophozoite	Decreased: CD40 ICAM-1 CD80 CD83 CD86		Suppressed DC ability to induce T cell activation DC suppression mediated by CD36 binding	CD36 ligation by *PfEMP1*
Urban et al, (71)	Monocyte-derived	ITO/C24 MC	Whole trophozoite	Decreased: HLA-DR CD83	Increased: IL-10 TNF-α Decreased: IFN-γ IL-4 IL-12 No change: TGF-β	Suppressed DC ability to induce T cell activation Co-incubation with CD40L does not restore DC function Apoptotic, but not necrotic cells have similar suppressive function	CD36 ligation by *PfEMP1*
Elliott et al, (104)	Monocyte-derived	ItG E8B CS2 3D7/upsCSBP1-KO	Whole trophozoite	Decreased: HLA-DR CD40 CD80 CD83 CD86	Decreased: IL-10 IL-12	pRBC lysate does not suppress LPS-mediated DC activation and can activate DCs High-dose pRBCs induce DC apoptosis Both CSA- and CD36-binding pRBCs inhibit LPS-mediated T cell proliferation	Parasite-to-DC ratio; *PfEMP1*-independent
Mukherjee and Chauhan, (105)	Monocyte-derived	3D7	Whole trophozoite	Increased: HLA-DR CD80 Decreased: CD86	Increased: TNF-α IL-6 IL-12 No change: IL-10	Stimulate CD4^+^ T cells to produce IFN-γ, IL-5, and IL-10	Phosphorylation of p38MAPK
			Free merozoite	Decreased: HLA-DR CD80 CD83 No change: CD86	Increased: IL-10 Decreased: IL-12 No change: TNF-α IL-6	Decreased phosphorylation of p38MAPK Stimulate CD4^+^ T cells to produce IL-10, low amounts of IFN-γ	Modulation of IL-10/IL-12 secretion via altering ERK1/2 signaling
Clemente et al. (106)	Monocyte-derived	3D7	Schizont lysate	Increased: CD86 No change: HLA-DR CD80	No change: IL-10 IL-12	Malaria-exposed DCs induce naive T cell differentiation into T_H_1 and T_REG_ subsets	
Götz et al. (85)	Monocyte-derived	3D7	Intact schizont	No change: HLA-DR CD80 CD86	No change: CCL2 CCL5 CXCL9 CXCL10 IL-1β IL-6 IL-10 TNFα		
***Bona fide*** **DCs^†^**							
Pichyangkul et al. (83)	Blood pDC (HLA-DR^+^CD123^+^)	TM267R GR MRU LA PH	Intact schizont Schizont lysate	Increased: CD86 CCR7 No change: CD40	Increased: IFN-α No change: TNF-α	Schizont-stimulated pDCs can induce γδ T-cell proliferation and IFN-γ production	
Wu et al. (107)	Blood cDC (HLA-DR^+^BDCA1^+^) Blood pDC (HLA-DR^+^BDCA2^+^)	3D7	Merozoite lysate		Increased: TNF-α IL-12p40 IFN-α	pDC-cDC cross-signaling is required for cytokine secretion by DCs	Cell-to-cell contact between DC subsets and other immune cells
Gowda et al. (108)	Blood cDC (HLA-DR^+^BDCA1^+^) Blood pDC (HLA-DR^+^BDCA4^+^)	3D7	Intact trophozoite	Increased: CD36	Increased: TNF-α No change: IL-12p40	DCs internalize CD36-binding pRBCs more efficiently	CD36 ligation by *PfEMP1*
Götz et al. (85)	Blood cDC (HLA-DR^+^BDCA1^+^) Blood pDC (HLA-DR^+^BDCA4^+^)	3D7	Intact schizont Schizont lysate	Increased: HLA-DR CD40 CD80 CD86	Increased: CCL2 CXCL9 CXCL10 IFN-α No change: CCL5 IL-1β IL-6 IL-10 TNF-α	Low ratios of pRBCs do not suppress DC activation by LPS pRBC-primed DCs induce T_H_1-like responses pDC-cDC cross-signaling is required for production of IFN-α, CXCL9 and CXCL10	NFκB- and PPARγ- independent

DC type, how DCs used in this study were derived; Parasite strain(s), P. falciparum laboratory strains used for stimulation; Stimulus type, parasite life stage used in this study and whether parasites were purified or processed. ^*^Monocyte-derived DCs, induced by co-incubating adherent precursors from whole blood with IL-4 and GM-CSF for 24 h (129).

† *Bona fide DCs, DCs which have full DC function after purification from donor tissues, without requiring any cytokine maturation*.

### MoDCs and Intracellular *P. falciparum* Blood-Stage Parasites

Initially, *P. falciparum* pRBCs were thought to suppress moDC function *in vitro* (103) as, when co-cultured with moDCs at a concentration of 100 parasites per DC, they impaired moDC activation via contact-dependent CD36-mediated mechanisms (103). In this study, DCs co-incubated with CD36-binding parasite lines displayed decreased expression of co-stimulatory markers CD40, CD54/ICAM-1, CD80, CD83, and CD86 in response to LPS stimulation, and had a low capacity for inducing allogeneic T cell proliferation (103). Co-incubation with non-CD36 binding parasite lines did not induce the same inhibition. However, a subsequent study found that a high ratio of pRBCs to DCs (100:1) inhibited LPS-induced DC maturation, cytokine production, and allogeneic T cell stimulation regardless of whether the parasite strain had a CD36-binding phenotype, and low doses of parasite (10:1) induced modest DC maturation and autologous T cell proliferation (104). This inhibition of LPS-induced DC maturation with high doses of pRBCs was co-incident with high levels of DC death *in vitro* (104).

Another study reported that a ratio of 10 pRBCs per moDC did not trigger upregulation of HLA-DR, CD83, or CCR7 on moDCs (132), contradicting the findings of Elliott et al. (104). However, the 100:1 ratio induced secretion of IL-1β, IL-6, IL-10, TNF-α, and upregulation of the pro-migratory chemokine receptor CXCR4 (132). Another report indicated that even at a ratio of 25 pRBCs per moDC, moDCs upregulated HLA-DR, CD40, CD80, and CD83 and secreted significantly higher levels of TNF-α, IL-6, and IL-10 (105). At higher pRBC-to-DC ratios, there was a corresponding increase in DC death (105).

Addition of CD40L to pRBC-stimulated moDCs enhanced HLA-DR and CD80 expression while CD86 expression was greatly reduced relative to CD40L alone (105). Secretion of TNF-α, IL-12, and IL-6 was also enhanced, while IL-10 secretion was unchanged relative to CD40L alone (105). In another study, exposure to schizont lysate triggered moDCs to upregulate CD86 but not CD80 or HLA-DR (106). These lysate-stimulated moDCs were capable of inducing allogeneic T cell differentiation into T_H_1 and regulatory T cells (T_REG_), both of which secreted high levels of IFN-γ. T_REG_ induced in this fashion also secreted high levels of IL-10 and TGFβ. Pre-incubating moDCs with parasite lysate did not affect their ability to undergo LPS-driven maturation (106). Lastly, moDCs stimulated with whole schizonts did not upregulate HLA-DR, CD80, or CD86, nor did they express cytokines or chemokines (Table 2) (85).

One explanation proposed by Elliott et al. (104) for the conflicting literature on the effect of pRBCs on moDC activation is that high ratios of pRBCs suppress DC function, while low ratios activate DCs (104), though in a recent study moDCs were not activated by stimulation with 3 pRBCs per DC (85). Alternately, variations in methodology are likely to contribute to some of the differences observed: different parasite strains and co-culture periods were used across all studies (Table 2). Moreover, the heterogeneity of moDC preparations can vary widely amongst different laboratories. Schizont lysate is also not a proxy for pRBCs as the lytic process produces a mixture of parasite membrane proteins, metabolites, and merozoites (107). The matter is further complicated by the multiple ways of defining “inhibition”: whether pRBCs truly block DC activation in response to an external stimulus, and which stimuli in particular are susceptible to this manner of inhibition. Alternatively, it must be clarified whether pRBCs induce higher levels of DC death.

In summary, while a dose-dependent relationship between pRBC dose and moDC inhibition is suggested, this relationship must be substantiated by further studies examining the individual roles that different parasite stimuli, strains, and methodological factors have on the final DC phenotype, preferably focusing on *bona fide* DCs in future studies. A more rigorous definition of moDCs and how “activation” and “inhibition” are defined in these cells, particularly given how different groups have used different activatory cytokine stimulation methods to drive DC-like cells to begin with, will be imperative to resolve existing conflicts.

### Monocyte-Derived DCs and Other *Plasmodium* Life Stages

Human DC responses to other *Plasmodium* life stages have been poorly investigated. Only one study has investigated moDC responses to *P. falciparum* merozoites (105). In this study, co-incubation with merozoites resulted in moDC secretion of TNF-α, IL-16, and large amounts of IL-10, despite no changes in costimulatory marker expression (105). Co-incubating merozoites with moDCs in the presence of CD40L induced high CD86 expression but no increase in other costimulatory surface markers (105). CD40L also induced merozoite-stimulated moDCs to produce high levels of IL-10 (105).

Likewise, only a single study to date has assessed moDC responses to *P. vivax* sporozoites (36). Prior to co-culture, moDCs were matured with TNF-α and LPS and primed, or not, with sporozoite extract. Primed moDC were more efficient than their unprimed counterparts at eliciting IFN-γ secretion and autologous T cell proliferation in DC-T cell co-cultures, and CD8^+^ T cells stimulated by primed moDCs had greater cytotoxic effector activity against infected HC04 hepatocyte lines (36). It is not yet known how DCs respond to other liver-stage parasites such as hypnozoites, exo-erythrocytic forms, or sexual-stage gametocytes.

### Interactions Between *Bona fide* Human DCs and *P. falciparum*

Due to the technical challenges in obtaining large numbers of viable *bona fide* human DCs from peripheral blood, relatively few studies have investigated direct interactions between *ex vivo* blood DCs from healthy donors and *P. falciparum* merozoites or pRBCs (Table 2). To date, studies have focused on BDCA-1^+^ cDC2 and pDC populations. None have examined the BDCA-3^+^ cDC1 subset, likely owing to the rarity of this population. Both merozoites and pRBCs have been shown to induce blood DCs to upregulate CD40, CD80, and CD86 (83, 85), and to secrete IFN-α (83, 85, 107), indicating that *P. falciparum* is capable of activating naïve DCs. Merozoites also triggered production of IL-12p40 and TNF-α (107). Additionally, a ratio of 3 pRBCs per DC resulted in upregulation of HLA-DR and increased expression of the chemokines CCL2, CXCL9, and CXCL10 (IP-10) (85), but did not trigger production of IL-1β, IL-6, IL-10, or TNF-α. Contrary to findings in moDCs, pRBCs did not suppress cytokine responses to LPS in *bona fide* DC, although this may be attributable to the lower pRBC-to-DC ratio used in this study (85). While the authors did not assess whether high doses of pRBCs modulated the ability of *bona fide* DCs to prime naïve T cells, as was shown for moDCs, *bona fide* DCs exposed to low doses of pRBCs were fully functional in their antigen presenting ability, inducing naive T cell proliferation and polarization toward an IFN-γ-producing T_H_1 phenotype (85). This does suggest, congruent with moDC studies (104, 106) and some CHMI studies (89, 91), that single, low-parasitaemia blood-stage infections of 10 pRBCs per DC or fewer, equivalent to 200 pRBCs/μL, may induce beneficial DC activation.

It is likely that cross-talk between different DC subsets plays an important role in immune responses to *P. falciparum*. Two studies that have examined this process indicate that DC cross-talk is required for production of TNF-α, IL-12p40 (107), IFN-α, CXCL9, and CXCL10 (also known as IP-10) (85) in response to pRBCs. In the context of antimalarial responses, DC activation appears to be contact-mediated and independent of IFN-α, although partially mediated by the TLR9 pathway (85, 107), expressed by just the pDC subset of human DCs. While cDCs alone are sufficient for inducing T cell activation to pRBCs, the presence of pDCs affects the ability of activated T cells to proliferate and produce cytokines (85). When a mixed culture of pDC and cDC was used in pRBC-primed autologous T cell stimulations, T cells trended toward reduced proliferation and production of IL-10, TNF-α, IFN-γ, and IL-5, but increased IL-2 secretion (85). It is possible that since the overall number of DCs for T cell stimulations was kept constant, reduced T cell activation was a consequence of the reduced proportion of cDCs.

These data highlight a need for future studies to investigate not only the individual roles of *bona fide* DC subsets in immunity to malaria, but also to consider the complexity of the immune response and the influence of cell-to-cell interactions. This should be reflected in the establishment of better *in vitro* models and cell-based systems that more realistically mimic the dynamic interactions and cell behaviors that occur over the course of an immune response *in vivo*.

### DC Interactions With Parasite by-Products

The cycle of parasite reproduction is fuelled by a range of host nutrients, not least of which is intraerythrocytic hemoglobin. Hemoglobin breakdown causes accumulation of toxic heme, which the parasite neutralizes by aggregating heme crystals into hemozoin (133). Hemozoin has been proposed to have both suppressive and activatory effects on DCs.

Initial studies reported that purified hemozoin induced CD1a, CD80, and CD83 upregulation and IL-12 secretion from moDCs, whereas monomeric heme and synthetic hemozoin (β-hematin) did not (134). However, these results were contradicted by a subsequent study demonstrating impaired upregulation of HLA-DR, CD40, CD80, CD83, ICAM-1, and CD1a in moDCs pre-incubated with *P. falciparum* hemozoin (135). These conflicting results may be due to the use of different hemozoin sources. Depending on the method of purification, parasite hemozoin can be contaminated with parasite proteins, nucleic acids, and other by-products that can activate alternate pathways. It is possible for even purified hemozoin to adhere to environmental contaminants after purification (133). The altered activity of hemozoin on treatment with phospholipase D (135) and DNAse (136) indicate that contamination with nucleic acids or other parasite metabolites is a likely explanation for the observed variations, particularly since hemozoin has been shown to be a carrier for *Plasmodium* DNA (136).

Murine models have been vital in establishing how hemozoin-malaria DNA complexes activate DCs. Murine Flt3-ligand-induced DCs (137) stimulated with hemozoin chelated to *P. falciparum* DNA secreted high levels of RANTES, IL-12, and TNF-α. In this system, hemozoin assisted in trafficking parasite DNA to intracellular endosomes, and activated DCs via a TLR9- and MyD88-dependent signaling pathway (136). Hemozoin in isolation bound strongly to the murine TLR9 ectodomain while β-hematin, a synthetic form of hemozoin, was unable to activate DCs in this study (136). A subsequent study in humans did observe CD80, CD83, and CXCR4 upregulation on human moDCs after β-hematin stimulation (132), but the β-hematin-induced DCs were unable to induce allogeneic T cell proliferation (132).

Uric acid, another toxic product of *P. falciparum* metabolism, has also been found to upregulate expression of CD80, CD86, and CD11c and to downregulate HLA-DR on purified human blood DCs (138). Uric acid also reportedly stimulated mast cells to produce high levels of the DC growth factor Flt3-L in mice (102). Interestingly, DNAse treatment of uric acid abrogated its activatory effects on DCs (138), similar to what is seen with hemozoin. While uric acid is known to drive inflammation during *Plasmodium* infection through activation of the inflammasome [reviewed in (139)], the role of the inflammasome in anti-malaria DC responses and activation has not been investigated.

Together these studies highlight an important role for *P. falciparum* DNA as an activatory ligand, particularly in activating pDCs and driving production of IFN-α (107). Since only the pDC subset in humans expresses TLR9 [reviewed in (140)], ligation of TLR9 by *P. falciparum* DNA and subsequent cytokine production by pDCs may be one of the primary mechanisms by which human DCs are activated by *Plasmodium* (107, 136). Cytoplasmic pattern recognition receptors for *Plasmodium* DNA, which are expressed in all DC subsets, may also play a prominent role in anti-*Plasmodium* interferon responses (141, 142). Of particular interest are the role of STING-dependent responses in DCs and their role in anti-malaria responses. Considering the wide distribution of *Plasmodium* DNA throughout the host during infection (143), the human DC response to malarial DNA *ex vivo* and *in vitro* is one of the key gaps in knowledge that remains unaddressed.

Murine studies have identified a number of other immunostimulatory *Plasmodium* products, but their role in DC activation has not yet been investigated. Glycosylphosphatidylinositol (GPI) molecules, membrane anchors for *Plasmodium* surface proteins, are known ligands for TLR2 (144), expressed on human cDCs (118). Parasite RNA is known to induce type I IFN via TLR7/MyD88-dependent signaling (145), and TLR7 is highly expressed on human pDCs (118), the major producers of type I IFN. Finally, microvesicles are small organelles of 1 μm of less in size, derived by blebbing of the plasma membrane, which are generated in high volumes during *Plasmodium* infection (146, 147). They can contain a range of parasite material, and are able to induce cytokine secretion from murine (148) and human (149) macrophages. In summary, considering the wide range of immunostimulatory molecules produced by *Plasmodium* spp., it remains interesting that the DC response to malaria is not always activatory. While further studies should continue to identify *Plasmodium* ligands that drive DC activation, this must be studied in combination with the factors that underlie DC suppression in malaria.

## DCs and *Plasmodium*: Outstanding Questions and Future Directions

Immunity to *Plasmodium* is complex, and many aspects of cellular immunity remain poorly understood, particularly the impact of malaria on DC function. A better understanding of DC responses to *Plasmodium* will provide insight into the low efficacy and relative short duration of protection of the current malaria vaccine RTS,S, as well as the slow acquisition of natural immunity in malaria-exposed individuals. One of the most important unresolved questions is one of DC “suppression”: namely, whether exposure to *Plasmodium* spp., particularly *P. falciparum*, inhibits the ability of DCs to initiate and orchestrate effective immune responses. Determining precisely how DCs are modulated by *P. falciparum* is crucial for understanding the development of immunity to malaria.

Some of the most interesting insights into the effects of malaria-induced DC impairment come from field studies that stratified patients by severity of infection. Studies that analyzed mild vs. severe malaria cases separately did not observe significant differences in DC phenotype between the groups (82, 102), suggesting that there may be a “tipping point” beyond which DC dysfunction is altered regardless of the severity of clinical presentation. This is underscored by the similarities in DC phenotype between natural exposure and CHMI. The latter are a naïve population, but during acute infection and for at least 24 h after treatment they exhibited similar DC phenotypes to those seen in naturally infected cohorts. Repeated infections could lead to sustained downregulation of DC function. Considering that successful induction of vaccine responses requires DC involvement, the failure of multiple malaria vaccines when transitioning from naïve populations to endemic populations (21, 150) may be due to impaired DC function in these endemic populations prior to vaccination.

**Figure 3:**
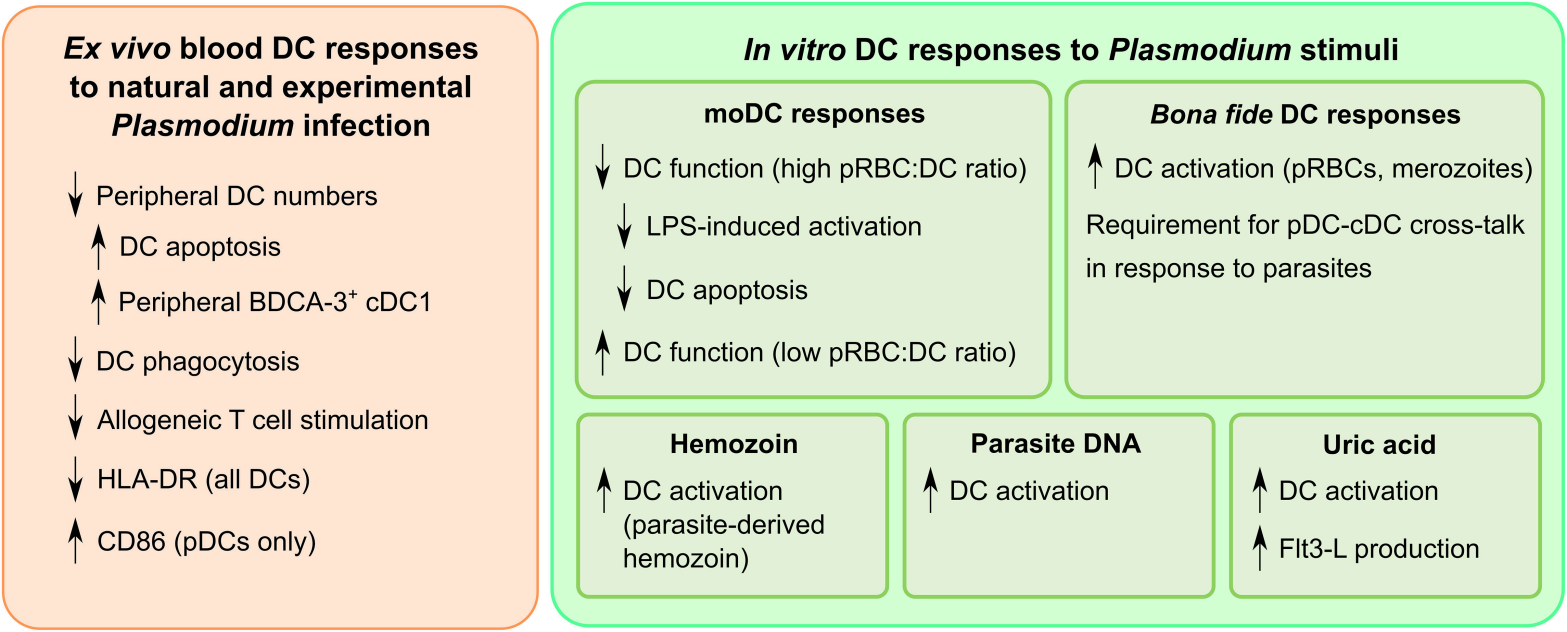
A summary of dendritic cell responses to *Plasmodium*. While the dendritic cell response is heterogeneous, certain trends are evident when examining the entirety of the current literature. Downregulation of dendritic cell function is commonly observed in field studies of infected humans. *In vitro* studies have yielded insight into the complexities of dendritic cell activation by *Plasmodium*, particularly the types of ligands that can trigger an inflammatory response.

Some valuable insights could be obtained by investigating differences in DC impairment between asymptomatic and symptomatic malaria cases. The systemic inflammation characteristic of symptomatic malaria undoubtedly contributes to DC dysfunction. While asymptomatic cases seem to experience similar loss of DC function short-term (87, 88, 99), it may be that they exhibit better recovery of DC function long-term. The phenotypic differences between high- and low-dose inoculation cohorts in CHMI studies (89, 91, 92) suggest that administering curative treatment while parasitaemia is still very low is also effective at limiting DC impairment. The similarities between naturally infected cohorts (Figure 3) also suggest that transmission intensity does not have a major impact on DC dysfunction beyond a certain threshold. Studies that follow the long-term effects of malaria exposure on DC function will be essential to clarify whether DC impairment persists after malaria elimination, especially in unstable transmission settings.

Variations in methodology have made it difficult to ascertain the effects *Plasmodium* parasites have on DCs *in vitro*. Particularly, care must be taken when stating that *P. falciparum* “suppresses” DC function *in vitro*: levels of activation lower than that seen in response to positive controls is not necessarily indicative of suppression. True suppression should be defined by an inability of DCs to become activated by known activatory stimuli, particularly pattern recognition receptor ligands. Analyses should also always account for increased cell death, which may result in false reports of suppression. Overall, the data indicates that DC function is not universally suppressed (Figure 3). Rather, *Plasmodium* appears to target specific pathways, among them the ones crucial for inducing naïve T cell proliferation (82, 103, 104), while still allowing DCs to polarize responses toward a T_H_1-like phenotype (85, 106). Research into whether the functionality of these T cells is affected relative to other pathogens which induce T_H_1 polarization will be important to understand the uniquely *Plasmodium* factors that delay the development of antimalarial immunity.

There also appears to be an important role for DC cross-talk between pDC and cDC subsets (85, 107). This has two crucial implications: first, it warrants further study of how individual DC subsets respond to *Plasmodium* spp. without grouping them into a monolithic conglomeration of HLA-DR^hi^Lin^−^ cells. In particular, elevation of the circulating BDCA-3^+^ cDC1 subset is a commonly reported phenomenon in field studies but, due to the low frequencies of this rare population, its function in malaria has not been extensively investigated. More rigorous strategies, including cell sorting, should be employed to study how purified subsets respond to malaria. Secondly, pDC cross-talk is essential for production of cytokines such as CXCL10 and IFN-α. CXCL10, also known as IP-10, is majorly implicated in malaria pathogenesis (151–153), and IFN-α has recently been shown to downregulate antimalarial immunity (154, 155). As pDC help is required for production of both of these cytokines, and pDCs are the major producers of IFN-α in malaria (65, 155), strategies to reduce pDC activation in malaria might be beneficial for the longevity of antimalarial immunity. Moreover, malarial DNA has proven to be a potent inflammatory ligand (107, 136), and since only pDCs express TLR9 in humans (118), detection of malarial DNA by pDCs may play a significant role in detrimental cytokine responses, making pDCs an ideal target for strategies to reduce pathogenicity.

Moreover, *ex vivo* and *in vitro* studies depict a complex effect of malaria on the pDC subset. Numbers of circulating pDCs were reduced during natural infection (83, 84, 86, 97, 99) and CHMI (92, 95), though it is unclear whether this is due to pDC death or sequestration. One murine study indicated that pDC could be infected with or endocytose parasites (156). Whether a similar situation exists for human pDC is not known, but it is plausible that large numbers of parasites within pDC could kill these cells, leading to lower circulating numbers. Malaria infection also triggers the upregulation of CCR7 on pDCs (83), suggesting that homing to lymphoid tissues is enhanced during infection. Thus, circulating pDCs may not be the subsets responding to infection, which could explain why pDC activation has not been reported in most field studies. Multiple other factors could also contribute to this perceived lack of pDC activation. Firstly, sustained parasitaemia in malaria-endemic regions may downregulate expression of activatory ligands on circulating pDCs. Secondly, non-conservative gating of pDCs may misrepresent the activation state of this DC population. Thirdly, no field studies to date have measured IFN-α production, which is a direct and functional read-out of pDC activation. *In vitro* studies have observed IFN-α production when pDCs were directly stimulated with parasite products (83, 85, 107). This reinforces the need to study pDC function during malaria to understand whether a loss of pDC numbers in the periphery is associated with a concomitant loss of function.

When interpreting existing literature, it must be kept in mind that the majority of studies have focused on pRBCs and only a minority have examined responses to extracellular forms of the parasite such as merozoites. Considering that DC responses change depending on the parasite life stage (85, 105, 107), even within the relatively limited scope of the blood stages, it will be vital to study the differences between responses to each life stage. Since it is unlikely that a single vaccine will be able to target the entire *Plasmodium* life cycle, understanding the type of responses induced by each life stage is essential for designing new therapeutic interventions to protect the host against damaging immunopathology. A two-pronged “big data” approach could be particularly informative, with the use of RNAseq or proteomics on the DC side to examine immune pathways induced by each parasite life stage, and conversely a proteomics or other—omics-based approach examining the potential immunogens expressed by each parasite life stage. In particular, understanding the pattern recognition receptor signaling pathways which are inhibited or activated by pRBCs would enable more targeted therapies to reverse their suppressive effects. This would inform functional studies, and therefore form a roadmap for vaccine strategies or other therapeutic approaches that could induce potent, long-lasting antimalarial immunity.

A primary caveat of the current literature on human DC responses to malaria, particularly in field studies of infected individuals, is that all studies have looked at circulating blood DCs. It may be that mature DCs migrate into the tissues while immature DCs remain in circulation. Therefore, care should be taken not to generalize the phenotype of these circulating DCs to the responses of liver, spleen or bone marrow tissue-resident DCs, which may have greater functional relevance. Understandably, obtaining tissue-resident cells directly from humans is difficult and ethically challenging. Thus, models such as humanized mice, which produce DCs functionally similar to those found in humans (57, 157), are a promising system to study DCs with a tissue-resident phenotype. While a humanized mouse that is able to support the entire *Plasmodium* life cycle is still out of reach, recent technological improvements have enabled development of models that allow study of immunity to specific life stages [reviewed in (158)]. For example, humanized liver mice could shed light on the elusive phenomenon of liver-stage immunity, while mice with humanized immune systems could provide better insight into cell-mediated mechanisms of protection against the blood stage. Development of a complete humanized mouse model for *Plasmodium* would be invaluable for human immunological research and vaccine development.

Organoids, miniature models of organ function, have proven useful in studying tissues such as the liver (159) and intestine (160). An intestinal organoid model has already been used to study transcriptomic regulation of another Apicomplexan with a complex life cycle, *Cryptosporidium* (161). Development of a splenic organoid could be used for development of functional tissue DCs and enable further study of blood-stage malaria, as well as other blood-borne diseases (162). Liver and skin organoids would also be invaluable for studying the pre-symptomatic phase of the life cycle, and aid development of a vaccine that confers sterile immunity.

Finally, data from *in vitro* studies using moDCs as a model may not be representative of the interactions between *Plasmodium* and steady-state DCs. Both moDCs and *bona fide* DCs show pro- and non-inflammatory responses to *Plasmodium* stimulation, but phenotypic and transcriptomic differences between them highlight that the moDC phenotype is pronouncedly different and may not necessarily be generalizable (125). It was previously thought that moDCs might be analogous to CD16^+^ DCs. However, recent findings outline that moDCs and CD16^+^ DCs exist as separate populations in the steady-state (124, 125), and while they may have convergent functions during inflammation, this has not yet been conclusively shown. Therefore, caution should be taken when describing DC-parasite interactions using results generated with moDCs: while it is likely that moDC-like APCs are generated during the course of *Plasmodium* infection, moDCs may not accurately reflect the behavior of steady-state DC populations.

This review has outlined many facets of DC function in malaria that are not well understood (Table 3). Firstly, DCs from naïve individuals appear to respond differently to each malaria life stage. These responses should be compared to those seen in exposed individuals for a better understanding of how prolonged malaria exposure affects immune recognition. Secondly, we must further develop an understanding of how the DC phenotypes we observe *ex vivo* and *in vitro* translate into effectiveness, duration, and quality of antimalarial responses. Including DC studies in vaccine trials would help to address which elements best describe a beneficial DC response. This should be supplemented by more studies of DCs in natural infection against comparable non-exposed donors. Thirdly, there is a need for better models to examine DC function in malaria. DC studies of the future should focus as much as possible upon *bona fide* DCs, and seek to develop new models that will permit a more in-depth study of how DC function is altered by the malaria parasite.

**Table 3:** Research priorities in DCs and malaria.

**Priority**	**Approach**
Understanding DC functionality to improve malaria vaccines	*In vitro* assays to understand which DC signaling pathways are activated or unaffected by malaria and exploit adjuvant technologies that target these pathways
	Incorporating DC functional assays into vaccine trials as a measure of vaccine relevance and functionality
Correlating DC function in malaria to protection	Controlled human malaria infection studies in naïve and previously exposed cohorts to understand how DC responses are altered by prior exposure and how this correlates with clinical immunity
	In-depth data analyses of how changes in DC phenotypes correlate with protective immune responses and/or overall clinical immunity
Understanding the mechanism of DC modulation by *Plasmodium* spp.	Development of small animal or *in vitro* models to assess human DC responses
	Thorough mapping of the functional and transcriptional changes that DC undergo upon encountering *Plasmodium* spp.
	Measuring DC responses to different *Plasmodium* life stages and determining which life stages have the greatest immunostimulatory potential to facilitate vaccine development

To conclude, the complexities of DCs make them a relatively understudied cell type in the context of malaria, where they have a potentially pivotal role in the regulation of antimalarial immunity. Many gaps in knowledge remain to be addressed, and there is a prominent need for novel technologies to bridge the gap. A deeper, more rigorous understanding of how *ex vivo* and *in vitro Plasmodium*-exposed DC phenotypes correlate with effective immunity, and the mechanisms that regulate DC interactions with *Plasmodium* will grant valuable insight into the acquisition of immunity, and form a basis for the development of better vaccines.

## Author Contributions

XZY wrote the first draft of the manuscript, which was reviewed and edited by RJL, JGB, and MOK. XZY and RJL prepared tables and figures. All authors have read and revised the manuscript.

### Conflict of Interest Statement

The authors declare that the research was conducted in the absence of any commercial or financial relationships that could be construed as a potential conflict of interest.
